# Molecular and morphological characterization of a first report of *Cactodera torreyanae* Cid del Prado Vera & Subbotin, 2014 (Nematoda: Heteroderidae) from Minnesota, the United States of America

**DOI:** 10.21307/jofnem-2021-093

**Published:** 2021-11-03

**Authors:** Zafar A. Handoo, Andrea M. Skantar, Sergei A. Subbotin, Mihail R. Kantor, Maria N. Hult, Michelle Grabowski

**Affiliations:** 1 Mycology and Nematology Genetic Diversity and Biology Laboratory, USDA, ARS, Northeast Area, Beltsville, MD 20705; 2 Plant Pest Diagnostic Center, California Department of Food and Agriculture, 3294 Meadowview Road, Sacramento, CA 95832; 3 Center of Parasitology of A.N. Severtsov Institute of Ecology and Evolution of the Russian Academy of Sciences; Leninskii Prospect 33, Moscow 117071, Russia; 4 Minnesota Department of Agriculture, Plant Protection Division, Saint Paul, MN 55155

**Keywords:** Cactodera torreyanae, Cyst nematode, Potato field

## Abstract

*Cactodera torreyanae* Cid del Prado Vera & Subbotin, 2014 cysts were discovered during a Pale Potato Cyst Nematode (PCN) survey conducted by Minnesota Department of Agriculture as part of the Animal and Plant Health Inspection Service (APHIS) efforts to survey states for the presence of PCN. The soil samples were collected from a potato field, located in Karlstad, Kittson County, Minnesota, USA. Two out of 175 vials submitted for identification to the Mycology and Nematology Genetic Diversity and Biology Laboratory (MNGDBL) contained few cysts and juveniles of *C. torreyanae*. Cysts were dark brown in color, lemon-shaped to elongated with distinct vulval cone. Vulva with denticles present around fenestra, cyst length to width ratio between 1.6 and 2.3 and anus distinct. The juveniles had rounded stylet knobs, some sloping slightly posteriorly. The molecular analysis included sequence and phylogenetic analysis of ITS rRNA, D2-D3 expansion segments of 28S rRNA and COI of mtDNA genes. The nematode species was identified by both morphological and molecular means as *Cactodera torreyanae*. To the best of our knowledge this represents the first report of *Cactodera torreyanae* from the United States and first report of this cyst nematode species from potato fields. Definite host plant for this nematode remains unknown.

In March 2020, a cyst nematode was discovered during a Pale Potato Cyst Nematode survey conducted by Minnesota Department of Agriculture as part of the Animal and Plant Health Inspection Service (APHIS) efforts to survey states for the presence of PCN. The soil samples were collected from a potato field, located in Karlstad, Kittson County, Minnesota, USA. The cyst samples were sent to the Mycology and Nematology Genetic Diversity and Biology Laboratory (MNGDBL) for identification purposes. Based on the results of morphological and molecular studies this nematode was identified as *Cactodera torreyanae* (Cid del Prado Vera and Subbotin, 2014). This species was originally described from saline soils of Texcoco, Mexico parasitizing *Sueda torreryana* (Cid del Prado Vera and Subbotin, 2014) and up to now this cyst nematode species was reported only from the type locality. The goal of this study is to provide a short morphological and molecular characterization of *C. torreyanae* from Minnesota, USA.

## Materials and methods

### Morphological study

**Figure 1:**
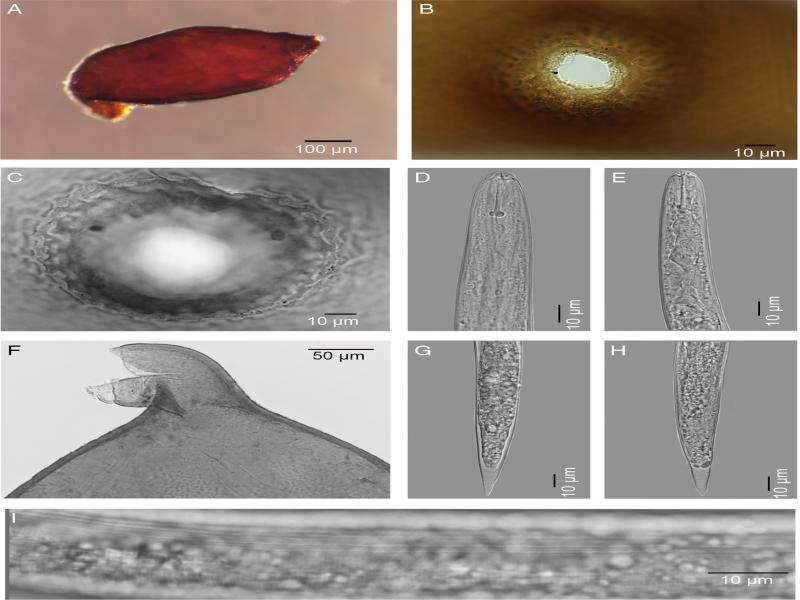
Photomicrographs of cysts, vulva cones and second-stage juveniles (J2) of *Cactodera torreyanae*. A: Entire cyst; B-C: Vulva cones; D-E: Anterior end of J2s; F: Cyst anterior end (arrow showing anterior end of J2 with stylet sticking out); G-H: Tails of J2s; I: Lateral field with 4 incisures for J2.

Cysts, second stage juveniles (J2), and eggs were obtained from two Minnesota soil samples collected from a location with the GPS coordinates: 48^o^57.248ʹN, 96^o^74.729ʹW. Juveniles were fixed in 3% formaldehyde and processed to glycerin by the formalin glycerin method (Golden, 1990; Hooper, 1970). Cysts contained viable eggs and second stage juveniles (J2) which were examined morphologically and molecularly for species identification. Observations of morphological characters critical for identification were cyst shape, color and nature of fenestration, cyst wall pattern, J2 stylet length, shape of stylet knobs, and shape and length of tail and hyaline tail terminus (Fig. 1). Photomicrographs of cyst vulval cones, females, and J2 were made with an automatic Nikon Eclipse Ni compound microscope using a Nikon DS-Ri2 camera. Measurements were made with an ocular micrometer on a Leitz DMRB compound microscope. All measurements are in micrometers. Mexican population of *C. torreyanae* was also used for molecular study.

### Molecular study

DNA was isolated from single juveniles disrupted in 20 µl Nematode Extraction Buffer. DNA extraction, amplification, purification of PCR products, cloning, and sequencing were performed as described in Skantar et al. (2012) and Subbotin (2021a). The following primer sets were used for PCR: the forward D2A (5′ – ACA AGT ACC GTG AGG GAA AGT TG – 3′) and the reverse D3B (5′ – TCG GAA GGA ACC AGC TAC TA – 3′) primers for amplification of the D2-D3 expansion segments of 28S rRNA gene; the forward TW81 (5′ – GTT TCC GTA GGT GAA CCT GC – 3′) and the reverse AB28 (5′ – ATA TGC TTA AGTT CAG CGG GT – 3′) primers for amplification of the ITS1-5.8S-ITS2 of rRNA gene, the forward JB3 (5′ – TTT TTT GGG CAT CCT GAG GTT TAT -3′) and the reverse JB4.5 (5′ – TAA AGA AAG AAC ATA ATG AAA ATG -3′) primers or the forward Het-coxiF (5′ – TAG TTG ATC GTA ATT TTA ATG G – 3′) and the reverse Het-coxiR (5′ – CCT AAA ACA TAA TGA AAA TGW GC – 3′) primers for amplification of the partial *COI* gene (Skantar et al., 2021; Subbotin, 2021a).

DNA sequencing was conducted by University of Maryland Center for Biosystems Research and Genewiz, Inc. ITS rRNA gene sequences were obtained from cloned amplicons; sequences of 28S and *COI* genes were obtained directly from PCR products. New sequences were submitted to GenBank under the following accession numbers: ITS rRNA gene (MZ753794-MZ753798); 28S rRNA gene (MZ753815-MZ753817, MZ773251) and *COI* gene (MZ753804, MZ753805, MZ773253, MZ773254).

The newly obtained sequences of the D2-D3 of 28S rRNA, ITS rRNA, *COI* genes were aligned using ClustalX 1.83 with corresponding published gene sequences (Cid del Prado Vera and Subbotin, 2014; Escobar-Avila et al., 2021; Li et al., 2021; Skantar et al., 2021). Sequence alignment was analyzed with Bayesian inference (BI) using MrBayes 3.1.2 (Ronquist and Huelsenbeck, 2003) under the GTR + G + I model and with PAUP* 4b10 (Swofford, 2002) as described by Subbotin (2021b).

## Results and Discussion

### Measurements and description

Measurements of second-stage juveniles from Minnesota (*n* = 7) included lengths of body (range = 421–510 μm, mean = 464.6 μm), stylet well developed (22.5–23.0 μm, 22.6 μm) with rounded basal knobs, tail (35.0–52.0 μm, 41.0 μm), and hyaline tail terminus (18.0–20.0 μm, 18.9 μm). The lateral field had four distinct lines. Shapes of the tail, tail terminus, and stylet knobs were also consistent with those of Mexican *C. torreyanae.* The cysts (*n* = 2) were lemon shaped, dark brown in color and dark brown in color, circumfenestrate, cone top with denticles present. Cyst wall with a wavy zigzag pattern in the middle. The fenestra length = 25.0, 32.5 μm, fenestra width = 28.0, 35.0 μm and the vulva slit to anus distance = 35.0, 37.5 μm. Morphometrics and morphology of cysts were consistent with those of Mexican *C. torreyanae* (Cid del Prado Vera and Subbotin, 2014) except for presence of denticles in the Minnesota population. Morphometrically, this species is also similar to *Cactodera weissi* (Steiner, 1949) Krall & Krall, 1978 in having small rounded to lemon shaped cysts with a circumfenestrate distinct vulval cone and in the J2 body and tail lengths and rounded stylet knobs. No males were found.

### Molecular characterization

**Figure 2:**
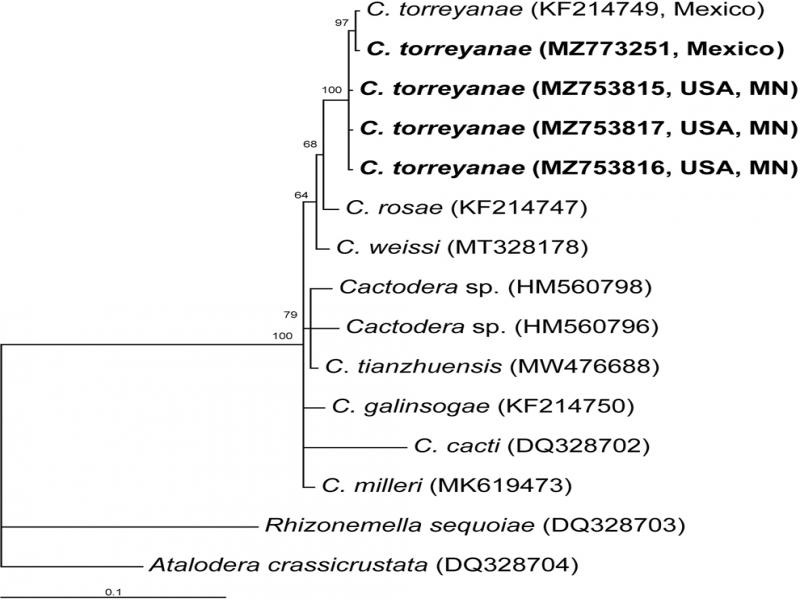
Phylogenetic relationships within the genus *Cactodera* as inferred from Bayesian analysis using the D2-D3 of 28S rRNA gene sequences under the GTR + I + G model. Posterior probabilities are given for appropriate clades. New sequences obtained in the present study are indicated in bold.

**The D2-D3 expansion segments of 28S rRNA gene.** The alignment was 722 bp in length and contained 13 *Cactodera* sequences, including four new sequences of *C. torreyanae* and two sequences of the outgroup taxa. Phylogenetic relationships within the genus *Cactodera* are given in Figure 2. The Minnesota *C. torreyanae* sequences clustered together (PP = 100%) with the sequences of the Mexican population of this species. The difference between the Minnesota and Mexican sequences was 0.1% (1 bp).

**Figure 3:**
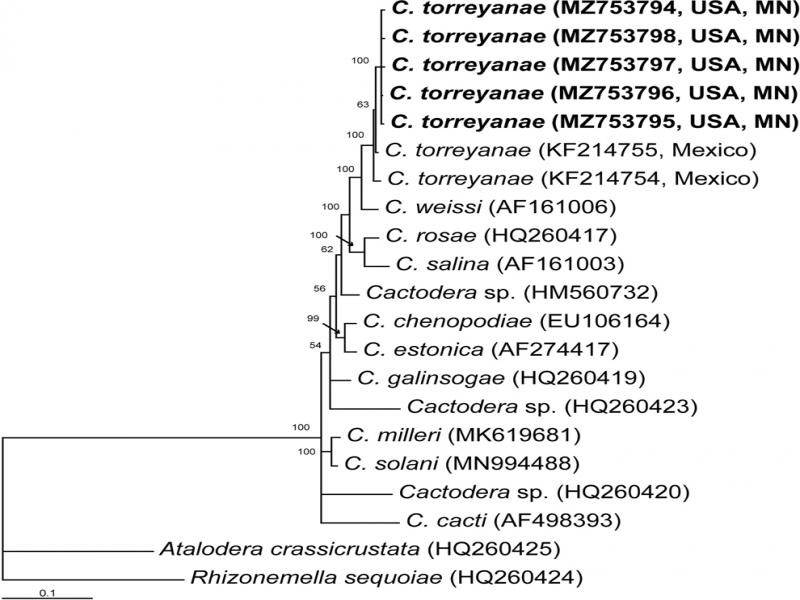
Phylogenetic relationships within the genus *Cactodera* as inferred from Bayesian analysis using the ITS rRNA gene sequences under the GTR + I + G model. Posterior probabilities are given for appropriate clades. New sequences obtained in the present study are indicated in bold.

**The ITS rRNA gene.** The alignment was 1030 bp in length and contained 19 *Cactodera* sequences, including five new sequences of *C. torreyanae* and two sequences of the outgroup taxa. Phylogenetic relationships within the genus *Cactodera* are given in Figure 3. The Minnesota *C. torreyanae* sequences clustered together (PP = 100%) with the sequences of the Mexican population of this species. The difference between the Minnesota and Mexican sequences was 0.7 to 1.4% (6–13 bp).

**Figure 4:**
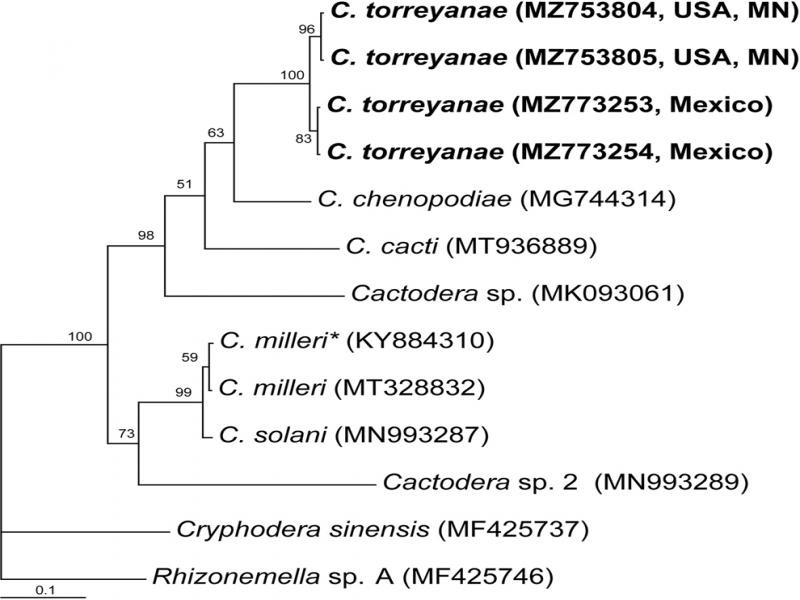
Phylogenetic relationships within the genus *Cactodera* as inferred from Bayesian analysis using the *COI* gene sequences under the GTR + I + G model. Posterior probabilities are given for appropriate clades. New sequences obtained in the present study are indicated in bold. *- identified as *C. torreyanae* in the GenBank.

**The** ***COI*** **gene.** The alignment was 452 bp in length and contained 11 *Cactodera* sequences, including four new sequences of *C. torreyanae* and two sequences of the outgroup taxa. Phylogenetic relationships within the genus *Cactodera* are given in Figure 4. The Minnesota *C. torreyanae* sequences clustered together (PP = 100%) with the sequences of the Mexican population of this species. The difference between the Minnesota and Mexican sequences was to 1.5% (6 bp). The difference between *C. milleri* and *C. solani* sequences was 1.1% (4 bp).

Based upon this collective morphological and molecular data, we identify this nematode as *Cactodera torreyanae.* The closest species morphologically and molecularly is *C. weissi* but was distinguishable based upon direct comparison of ITS rRNA, 28S rRNA and *COI* gene sequences. To our knowledge this is the first report of the *Cactodera torreyanae* from United States and first report of this cyst nematode species from a potato field. Definite host plant for this nematode remains unknown.
